# Genomic and Metabolic Hallmarks of SDH- and FH-deficient Renal Cell Carcinomas

**DOI:** 10.1016/j.euf.2021.12.002

**Published:** 2022-03-11

**Authors:** Angela Yoo, Cerise Tang, Mark Zucker, Kelly Fitzgerald, Renzo G. DiNatale, Phillip M. Rappold, Kate Weiss, Benjamin Freeman, Chung-Han Lee, Nikolaus Schultz, Robert Motzer, Paul Russo, Jonathan Coleman, Victor E. Reuter, Ying-Bei Chen, Maria I. Carlo, Anthony J. Gill, Ritesh R. Kotecha, A. Ari Hakimi, Ed Reznik

**Affiliations:** a Urology Service, Department of Surgery, Memorial Sloan Kettering Cancer Center, New York, NY, USA; b SUNY Downstate Health Sciences University, Brooklyn, NY, USA; c Computational Oncology, Memorial Sloan Kettering Cancer Center, New York, NY, USA; d Physiology, Biophysics and Systems Biology Graduate Program, Weill Cornell Medicine, New York, NY, USA; e Genitourinary Oncology Service, Department of Medicine, Memorial Sloan Kettering Cancer Center, New York, NY, USA; f Center for Molecular Oncology, Memorial Sloan Kettering Cancer Center, New York, NY, USA; g Department of Pathology, Memorial Sloan Kettering Cancer Center, New York, NY, USA; h Sydney Medical School, University of Sydney, Sydney, Australia; i Cancer Diagnosis and Pathology Group, Kolling Institute of Medical Research, Royal North Shore Hospital, St. Leonards, Australia; j NSW Health Pathology, Department of Anatomical Pathology, Royal North Shore Hospital, St. Leonards, Australia

**Keywords:** SDHRCC, FHRCC, SDH, FH, Renal cell carcinoma, Metabolism, Cancer genomics

## Abstract

**Background:**

Succinate dehydrogenase-deficient and fumarate hydratase-deficient renal cell carcinomas (SDHRCC and FHRCC) are rare kidney cancers driven by loss of TCA cycle enzymes.

**Objective:**

To define and compare the genomic and metabolomic hallmarks of SDHRCC and FHRCC.

**Design, setting, and participants:**

We analyzed SDHRCC and FHRCC tumors with either immunohistochemical evidence of loss of protein expression or genomically confirmed biallelic inactivation of *SDHA/B/C/D/AF2* or *FH*.

**Outcome measurements and statistical analysis:**

Somatic alterations were identified using clinical pipelines, with allele-specific copy number alterations (CNAs) identified using FACETS. Mass spectrometry–based metabolomic profiling was performed on available SDHRCC and FHRCC tumors.

**Results and limitations:**

Tumors were analyzed for 42 patients (25 FHRCC, 17 SDHRCC). In the germline analysis, 16/17 SDHRCCs harbored a germline alteration in *SDHB*, whereas only 17/22 FHRCCs had pathogenic germline *FH* variants. SDHRCCs had a lower mutation burden (*p* = 0.02) and CNA burden (p = 0.0002) than FHRCCs. All SDHRCCs presented with deletion of chromosome 1p (overlapping *SDHB*), whereas FHRCCs demonstrated high but not ubiquitous loss of 1q (*FH* locus). Both SDHRCCs and FHRCCs exhibited significant idiopathic accumulation of the metabolite guanine. FHRCC tumors had elevated levels of urea cycle metabolites (argininosuccinate, citrulline, and fumarate), whereas SDHRCC tumors had elevation of numerous acylcarnitines. These characteristic metabolic changes allowed identification of a previously unrecognized SDH-deficient RCC.

**Conclusions:**

Despite sharing similar genetic etiology, SDHRCC and FHRCC represent distinct molecular entities with unique genetic and metabolic abnormalities.

**Patient summary:**

Kidney cancers driven by loss of the gene encoding either the succinate dehydrogenase or fumarate hydratase enzyme are rare. We sought to define and compare the genetic and metabolic features of these cancer entities.

## 1. Introduction

Mutations in protein subunits of succinate dehydrogenase (*SDHA/SDHB/SDHC/SDHD* and assembly factor *SDHAF2*) and fumarate hydratase (encoded by the single gene *FH*) lead to rare forms of renal cell carcinoma (RCC; denoted by SDHRCC and FHRCC, respectively) [1]. SDH and FH catalyze consecutive reactions in the tricarboxylic acid (TCA) cycle, and SDH also encodes Complex II of the mitochondrial electron transport chain, which is responsible for oxidation of FADH_2_ [2]. Both SDHRCC and FHRCC are rare: it is speculated that SDHRCC constitutes 0.05–0.2% of all RCC diagnoses [3], while FHRCC is comparatively more common but still rare, accounting for 0.5–4.35% of all RCC diagnoses [4].

Owing to their rarity, comprehensive evaluation of the genetic and metabolic features of SDHRCC and FHRCC tumors has been limited [41]. In vitro data for mouse hepatocyte cell lines with silencing of *FH* or *SDHA* suggest that complete loss of either enzyme produces a block in the TCA cycle and consequent accumulation of upstream metabolic intermediates (succinate in SDHRCC and fumarate in FHRCC) [5]. Both fumarate and succinate can act as potent inhibitors of α-ketoglutarate-dependent dioxygenases including DNA demethylases [6], and both have been implicated as drivers of hypermethylation phenotypes seen in RCC, paragangliomas, and pheochromocytomas [7,8], suggesting that loss of either of the two enzymes may converge on a common phenotype. RCC patients with a germline *FH* or *SDH* mutation are predisposed to developing additional syndromic features; FHRCC patients can develop cutaneous and uterine leiomyomas [9] comprising a genetic syndrome called hereditary leiomyomatosis and RCC (HLRCC); SDHRCC patients have a higher risk of developing pheochromocytomas/paragangliomas [10] and gastrointestinal stromal tumors [11].

The functional similarity of the SDH and FH enzymes and the similar downstream epigenetic changes caused by accumulation of the associated oncometabolites suggest that SDHRCC and FHRCC may bear genomic and metabolic similarities. Three recent studies, including one by our group, have described several genetic hallmarks of FHRCC, including a characteristically low tumor mutation burden, distinct tumor microenvironments characterized by CD8^+^ T-cell infiltration with PD-L1 expression in tumors, and a highly depleted and mutant mitochondrial genome [7,12,13]. By contrast, prior reports characterizing SDHRCC tumors largely focused on clinicopathologic hallmarks of the disease and genetic analyses of specific *SDH* mutant alleles, but did not describe the full genomic landscape of SDHRCC [4,14,15]. Hence, comprehensive molecular and metabolomic profiling of SDHRCC tumors has been limited [16].

To characterize and compare the molecular characteristics of SDHRCC in relation to FHRCC, we assembled a multi-institutional genomically defined cohort of SDHRCC and FHRCC tumors. We describe two novel discoveries. First, we report that SDHRCCs exhibit a recurrent pattern of somatic genetic alterations, most prominently characterized by near-universal loss of heterozygosity on chromosome 1p. Second, by applying metabolomic profiling to a subset of SDHRCC and FHRCC cases, we identify characteristic metabolic alterations associated with TCA-cyclemutated kidney cancers, including large accumulation of the metabolite guanine. We then apply metabolomic profiling as an exploratory exercise in a previously cohort of unclassified RCC tumors and identify a previously unrecognized SDHRCC patient within the cohort.

## 2. Patients and methods

### 2.1. MSK-IMPACT targeted sequencing

For patients for whom targeted sequencing was carried out, tumor and matched normal samples were sent for targeted sequencing using our previously validated sequencing panel (MSK-IMPACT), of which three versions exist, targeting 341, 410, or 468 actionable cancer-associated genes, respectively [17]. DNA from tumor and matched normal blood specimens were extracted from each patient to create barcoded DNA libraries. Using the captured DNA, all coding exons of the target genes, as well as a subset of polymorphic loci (for copy number analysis) were sequenced. Deep sequencing was performed at an average coverage of 500× to 1000×. After alignment to the reference human genome, somatic alterations (including missense mutations, small insertions and deletions, structural rearrangements, and DNA copy number alterations [CNAs]) were identified using a bioinformatics pipeline described in detail previously [17]. The OncoKB oncology knowledge database was used to annotate mutations to assess oncogenicity [18]. All patients were consented to their respective institutional tissue banking protocols for secondary analysis.

### 2.2. Whole-exome sequencing, processing, and mutation analysis

Whole-exome sequencing (WES) samples (from 8 SDHRCC patients; 2 with matched normal samples and 6 unmatched) were processed and analyzed using the TEMPO pipeline (v1.3, https://ccstempo.netlify.app/). In brief, demultiplexed FASTQ files were aligned to the b37 assembly of the human reference genome from the GATK bundle using BWA mem (v0.7.17). Aligned reads were converted and sorted into BAM files using samtools (v1.9) and marked for PCR duplicates using GATK MarkDuplicates (v3.8-1). Somatic mutations (single nucleotide variants and small insertions and deletions) were called in tumor-normal pairs using MuTect2 (v4.1.0.0) and Strelka2 (v2.9.10), and structural variants were detected using Delly (v0.8.2) and Manta (v1.5.0). Variants were annotated and filtered for recurrent artifacts and false positives using methods as previously described [19]. As with the IMPACT samples, the OncoKB knowledge base was used to identify oncogenic alterations [18]. The tumor mutational burden (TMB) was defined as the number of nonsynonymous mutations in canonical exons per megabase. For copy number analysis, as for the targeted sequencing data, we used FACETS v0.5.6 as described in detail below.

### 2.3. Copy number and mutation analysis

For zygosity determination, genome-wide total and allele-specific DNA copy number, purity, and ploidy were calculated via FACETS v0.5.6 [20]. The expected number of copies for each mutation was generated based on the variant allele fraction observed and local ploidy [21]. Cancer cell fractions were calculated using a binomial distribution and maximum likelihood estimation normalized to produce posterior probabilities [22]. We then assessed what fraction of samples had either gains or loss of heterozygosity (LOH; either absolute loss of copy or copy-neutral LOH) at each cytoband in the genome, applying a clonality cutoff of 0.25. In generating the oncoprints, we restricted the gene set to genes that were mutated at least three times in our sample set, and classified mutation events (instances where a gene was altered in a patient) according to the type of mutation and whether there were multiple mutations. We compared TMB for FHRCC and SDHRCC by taking the total number of nonsilent somatic mutations across IMPACT-341 genes for patients in each subtype and applying the Wilcoxon test to assess whether the distributions were significantly different. To compare the fraction of genome altered (FGA), we computed the fraction of the sequenced portion of the genome (which varies depending on whether the sample is WES, whole-genome sequencing, or IMPACT) subject to either loss or gain of copy or copy-neutral LOH. We then compared FGA between FHRCC and SDHRCC using the Wilcoxon test.

### 2.4. Metabolomic profiling

After obtaining informed consent and approval from the Memorial Sloan Kettering Cancer Center (MSKCC) institutional review board, partial or radical nephrectomies were performed at MSKCC and tissue was stored at the MSK Translational Kidney Research Program. Samples were flash frozen and stored at −80 °C before metabolomic characterization. Clinical metadata were recorded for all tumor samples. Samples were thawed and extracted according to the standard Metabolon protocol to remove proteins, dislodge small molecules bound to proteins or physically trapped in the protein matrix, and recover a wide range of chemically diverse metabolites. Samples were then frozen, dried under vacuum, and prepared for liquid chromatography (LC)/mass spectrometry (MS).

The sample extract was split in two and reconstituted in acidic and basic LC-compatible solvents. Samples were eluted with a methanol/water gradient; the solvent contained 0.1% formic acid for the acidic extracts and 6.5 mM ammonium bicarbonate for the basic extracts. One aliquot was analyzed using conditions optimized for acidic positive ions and the other using conditions optimized for basic negative ions. The two aliquots were independently injected onto separate dedicated columns. The MS analysis alternated between MS and data-dependent MS/MS scans using dynamic exclusion. The LC/MS portion of the platform was based on a Waters ACQUITY UPLC and a Thermo-Finnigan LTQ-FT mass spectrometer, which had a linear ion-trap at the front end and a Fourier-transform ion cyclotron resonance mass spectrometer at the back end. Accurate mass measurements could be performed for ions with counts greater than 2 million. The average mass error was <5 ppm. Greater effort was required to characterize ions with a count of less than 2 million. Typically, fragmentation spectra (MS/MS) were generated in a data-dependent manner, but targeted MS/MS could be used if necessary, such as in cases of lower signal levels.

Data were extracted from the raw MS files, which were loaded into a relational database. The information was then examined and appropriate quality control limits were imposed. The Metabolon proprietary peak integration software was used to identify peaks, and results for components were stored in a separate data structure. Metabolites were compared to an in-house library of standards from Metabolon. Data for each of these standards were based on retention index, mass-to-charge ratio, and MS/MS spectra. These parameters for each feature for each compound in the metabolomic data were compared to analogous parameters in the library. As described by Evans et al. [23], compounds were identified on the basis of three criteria: retention index (RI) within 75 RI units of the proposed identification, mass within 0.4 m/s, and MS/MS forward and reverse matched scores. Each compound was corrected in run-day blocks by registering the medians to equal one and normalizing each data point accordingly. The data were subsequently log_2_ normalized. When metabolite levels were below the level of detection, the lowest measured abundance of that compound across all samples was imputed. All statistical tests were performed in R. Distributions were compared using the Wilcoxon test. All statistical analyses were two-sided and Benjamini-Hochberg correction was applied to *p* values.

## 3. Results

### 3.1. Cohort characteristics

We identified 83 RCC patients with a presumed diagnosis of FHRCC or SDHRCC from MSKCC and through a multi-institutional cohort collaboration [14]. Patients were included if they had sequencing data and met molecular criteria (biallelic loss of *FH* or *SDHA/SDHB/SDHC/SDHD/SDHAF2*) or immunohistochemistry (IHC) criteria (loss of FH expression and/or presence of 2-succino-cysteine positivity [24,25], or loss of SDHB expression [26]; Supplementary Fig. 1 and Supplementary Table 1). The final cohort consisted of 25 FHRCC and 17 SDHRCC patients (Table 1).

SDHRCC and FHRCC patients presented with disease at a young age (median age 32 yr for SDHRCC, 47 yr for FHRCC; *p* = 0.03) and had comparable gender ratios. Other demographic and clinical information is provided in Supplementary Table 1. Six patients from our institution presented with localized disease (1 of whom went on to develop metastatic disease), while three SDHRCC patients were diagnosed with metastatic disease at initial presentation. Four SDHRCC patients received various systemic therapies; more information is provided in Supplementary Table 2.

### 3.2. Distinct and recurrent CNAs are the primary events in the evolution of SDHRCC and FHRCC

**Fig. 1 –:**
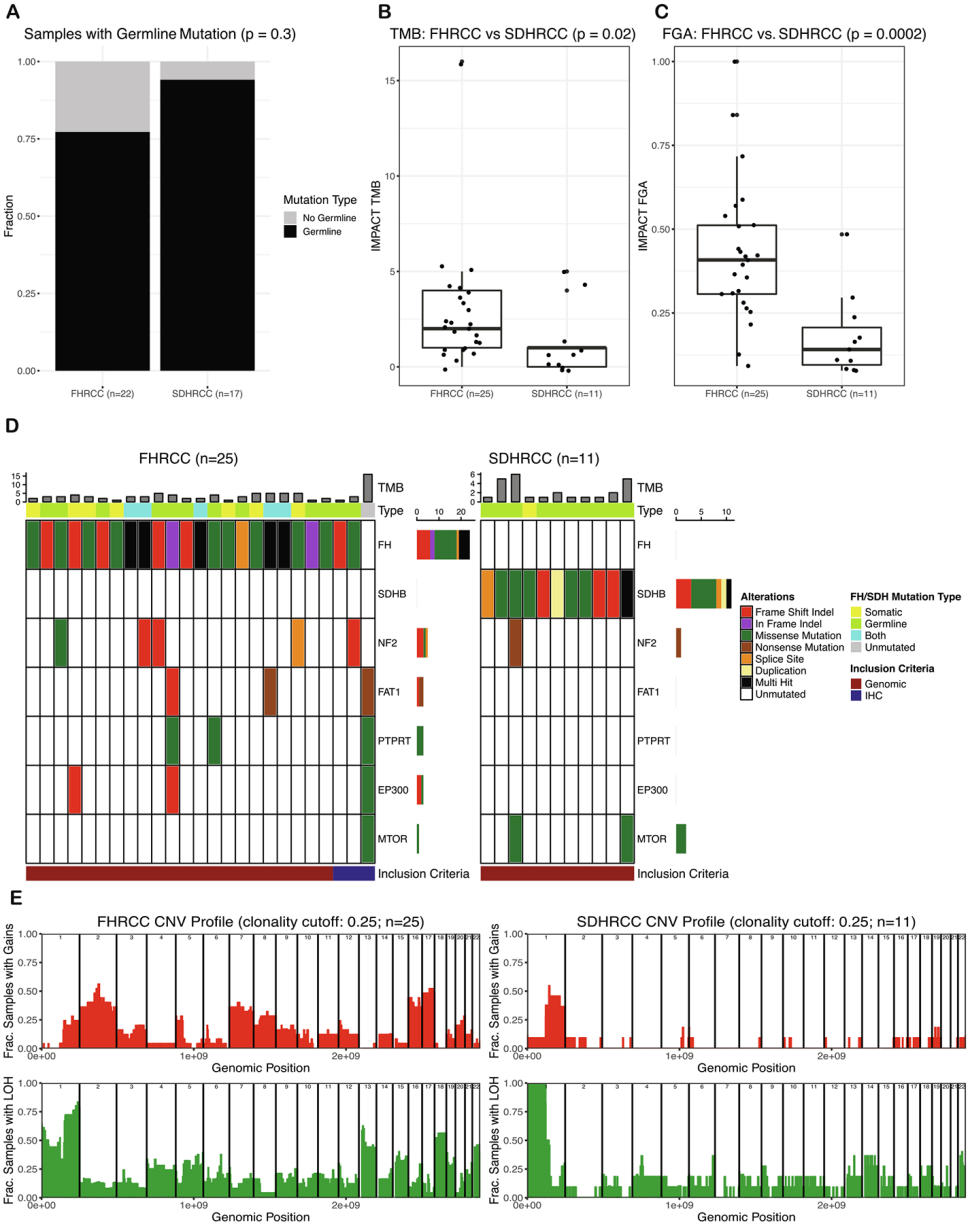
Genomic analysis and comparison of FHRCC and SDHRCC. (A) Comparison of the incidence of germline mutations in the FHRCC and SDHRCC cohorts. SDHRCC tumors are more likely to harbor pathogenic germline variants. Only 22/25 patients with FHRCC consented to germline mutation analysis. (B) FHRCC tumors have a higher tumor mutational burden and (C) fraction of the genome altered. (D) Oncoprint displaying recurrent (genes mutated in ≥3 patients) somatic mutations in FHRCC and SDHRCC tumors. Within the oncoprint, 2/11 SDHRCC and 10/25 FHRCC samples had recurrent mutations. However, 5/11 SDHRCC (45%) and 21/25 (84%) FHRCC samples exhibited a mutation when evaluating any occurrence of at least one somatic mutation. (E) Copy-number profiles for FHRCC and SDHRCC. The top panels indicate the fraction of samples with gains. The bottom panel indicates the fraction of samples with LOH (including copy-neutral LOH). SDHRCC demonstrates universal LOH on chromosome 1p, whereas FHRCC often demonstrates LOH on chromosome 1q. RCC = renal cell carcinoma; FHRCC = FH-deficient RCC; SDHRCC = SDH-deficient RCC; LOH = loss of heterozygosity; TMB = tumor mutational burden; FGA = fraction of the genome altered; IHC = immunohistochemistry; CNA = copy number alteration; Frac. = fraction.

To assemble a genomically defined cohort of SDHRCC with sufficient power to identify recurrent and discriminatory molecular features, we aggregated tumors profiled by different sequencing modalities into a multi-institutional cohort. Mutations and CNAs from all patients are listed in Supplementary Table 3 and Supplementary Table 4. We identified biallelic alterations in all 17 patients with SDHRCC, whereas biallelic alterations were evident in 22/25 FHRCC patients. Of the 17 SDHRCC patients, 16 had pathogenic germline mutations; one SDHRCC patient for whom whole-genome sequencing was carried out had biallelic *SDHB* inactivation via somatic mutation and concomitant LOH (Fig. 1A). Interestingly, the somatic mutation observed in this patient (at amino acid 242) has previously been reported as a germline mutation, suggesting evolutionary convergence on deleterious alleles [27]. FHRCC patients demonstrated a lower rate of germline alterations: 17/22 FHRCC patients who had consented to germline analysis had pathogenic germline mutations. This suggests that the majority of SDHRCC and FHRCC cases are associated with germline alterations, but both can arise through purely somatic biallelic inactivation.

We next evaluated whether secondary somatic mutations in other cancer-associated genes present in SDHRCC and FHRCC tumors converged on common molecular pathways. FHRCC tumors were in general more highly mutated than SDHRCC tumors, with a significantly higher TMB (*p* = 0.02; Fig. 1B), and had significantly more chromosomal CNAs (FGA; *p* = 0.0002, Wilcoxon test, Fig. 1C). The most commonly mutated genes in FHRCC patients, after *FH,* were *NF2* (*n* = 5), *FAT1* (*n* = 3), *PTPRT* (*n* = 3), and *EP300* (*n* = 3; Fig. 1D). Four of the five *NF2* mutations present were approximately clonal (cancer cell fraction >0.75), while two out of three *FAT1*, *PTPRT,* and *EP300* mutations each were approximately clonal. Most of these mutations (all *NF2* mutations, 2/3 *FAT1* mutations, 2/3 *EP300* mutations, and 1/3 *PTPRT* mutations) were accompanied by LOH and therefore represent bona fide biallelic loss of these tumor suppressors. SDHRCC tumors tended to have few, if any, accompanying somatic mutations, with a single case showing a subclonal *NF2* mutation. Both SDHRCC and FHRCC tumors lacked a significant number of mutations in common ccRCC-associated genes, including *PBRM1* (*n* = 1), *SETD2* (*n* = 0), *TERT* (*n* = 1), and *TP53* (*n* = 1).

In contrast to somatic mutations, SDHRCC and FHRCC tumors demonstrated numerous large-scale CNAs (Fig. 1E). Although SDHRCC patients generally had fewer CNAs, all exhibited LOH (either copy number loss or copy-neutral LOH) on chromosome 1p, overlapping with the *SDHB* locus. Furthermore, manual inspection confirmed that all six SDHRCC patients who underwent WES without matched normal (eg, blood) tissues also demonstrated LOH on chromosome 1p. In ~50% of cases, 1p LOH was accompanied by single-copy amplification of chromosome 1q. Besides chromosome 1, there were no highly recurrent CNAs among SDHRCC patients. By contrast, FHRCC patients usually—although not always—had LOH on chromosome 1q, particularly in the region overlapping *FH*. FHRCC tumors showed a higher incidence of other CNAs, including gains on chromosomes 16 and 17, which occurred in >40% of FHRCC patients. FHRCC patients were also more likely to have gains on chromosome 2, with approximately one-third having this alteration, while only 1/11 SDHRCC patients had a gain on chromosome 2.

### 3.3. Distinct metabolic phenotypes beyond the TCA cycle underlie SDHRCC and FHRCC

**Fig. 2 –:**
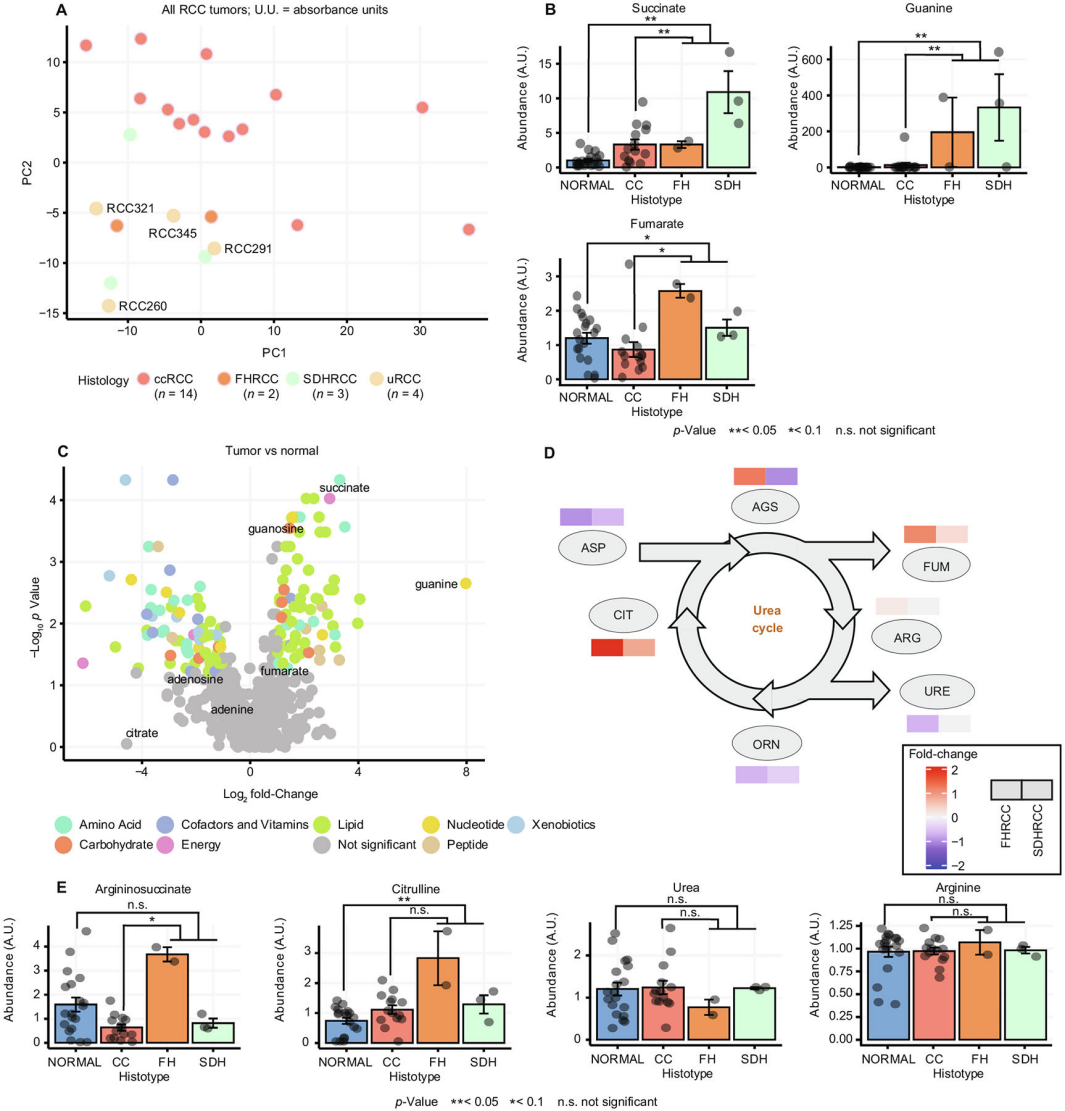
Metabolomic analysis and comparison of FHRCC and SDHRCC. (A) Principal component analysis plot of ccRCC (*n* = 14), unclassified RCC (*n* = 4), SDHRCC (*n* = 3), and FHRCC tumors (*n* = 2). The unclassified RCC tumors as well as the SDHRCC and FHRCC tumors cluster away from the ccRCC tumors. (B) Barplot showing levels of succinate, fumarate, and guanine in normal kidney tissue and ccRCC, FHRCC, and SDHRCC tumors. (C) Volcano plot of metabolites that were elevated in SDHRCC/FHRCC tumors compared to normal tissue, including succinate and guanine. (D) Urea cycle metabolites (argininosuccinate and citrulline) are more elevated in FHRCC than in SDHRCC when compared to normal tissue. (E) Barplot showing levels of argininosuccinate, urea, citrulline, and arginine in normal kidney tissue and ccRCC, FHRCC, and SDHRCC tumors. Argininosuccinate and citrulline were uniquely elevated in FHRCC, but urea and arginine were not. RCC = renal cell carcinoma; FHRCC = FH-deficient RCC; SDHRCC = SDH-deficient RCC; ccRCC = clear-cell RCC; PC1 = principal component 1; PC2 = principal component 2; A.U. = absorbance unit; AGS = argininosuccinate; FUM = fumarate; ARG = arginine; URE = urea; ORN = ornithine; CIT = citrulline; ASP = aspartate.

Complete loss of SDH and FH in vitro leads to accumulation of the upstream metabolites succinate and fumarate, respectively, and disruption of metabolic pathways dependent on normal TCA cycle function. To study the metabolic alterations underlying SDHRCC and FHRCC in vivo, we identified three SDHRCC and two FHRCC tumors from our genomic cohort with suitable fresh-frozen tissue for metabolomic profiling. For comparison, we also profiled 14 ccRCC samples, four unclassified RCC (uRCC) samples, and 19 matched normal kidney tissue samples. Principal component analysis (PCA) separated both FHRCC and SDHRCC from ccRCC, indicating that these entities broadly display unique metabolic phenotypes relative to ccRCC (Fig. 2A).

Of the eight TCA cycle metabolites quantified, succinate exhibited the largest increase in abundance compared to normal tissue when grouping FHRCC and SDHRCC samples together, but succinate accumulation was greater in SDHRCC (log_2_ fold-change 3.41; *p* = 0.001, *q* = 0.08; Supplementary Table 5, Fig. 2B) than in FHRCC (log_2_ fold-change 1.69; *p* = 0.02, *q* = 0.34). By contrast, fumarate levels were uniquely elevated in FHRCC, consistent with the position of SDH upstream of FH. Despite complete loss of *FH,* FHRCC samples demonstrated only an approximately twofold increase in fumarate levels that did not reach statistical significance (*p* = 0.02, *q* = 0.34), in contrast to the ~50-fold increase observed when *FH* is ablated in vitro [28]. Owing to the limitations of metabolomic profiling of bulk tumor samples, we were unable to determine if this effect was due to the presence of infiltrating stromal/immune cells with normal fumarate levels or upregulation of mechanisms for fumarate detoxification in vivo, or if FHRCC tumors themselves have characteristically lower levels of fumarate than expected from cell-line data.

### 3.4. Guanine pools are elevated in both SDHRCC and FHRCC

We identified 152 metabolites (excluding succinate) with a statistically significant difference in abundance between FHRCC/SDHRCC and normal kidney (*q* < 0.2 and log_2_ fold-change >1; Fig. 2C). Of these, 12 metabolites (8%) also demonstrated a statistically significant difference in abundance relative to ccRCC tumors, suggesting they were specific adaptations associated with TCA cycle dysfunction. Among the metabolomic adaptations in SDHRCC and FHRCC, the purine derivative guanine had the largest fold-change (~250-fold; 7.97 log_2_ fold-change; *p* = 0.002, *q* = 0.04; Fig. 2B). Interestingly, extreme elevation of guanine in FHRCC and SDHRCC was accompanied by a smaller elevation in guanosine (1.57 log_2_ fold-change; *p* = 0.0002, *q* = 0.01), but no change in purine-derived adenine or adenosine, and no change in the catabolic product of guanine, xanthine. Importantly, guanine was also elevated ~20-fold relative to ccRCC tumors (*p* = 0.005, *q* = 0.11; Fig. 2B), indicating that this metabolic phenotype was specific to disruption of FH and SDH. A similar extent of guanine accumulation was observed in six tumor regions from an additional FHRCC tumor (Supplementary Table 6). The extremely high elevation of the guanine pool in SDHRCC and FHRCC suggests that loss of either FH or SDH may cause either overflow into free guanine from a peripheral pathway, or alternatively may prevent turnover of free guanine by enzymes that rely on an intact TCA cycle.

Although FHRCC and SDHRCC tumors displayed common metabolic alterations, we noted that the two entities nevertheless clustered separately on PCA, indicating that they are characterized by qualitatively different metabolic phenotypes. However, the small sample size for our data set (3 SDHRCC and 2 FHRCC samples) did not allow de novo discovery of statistically significant differences in metabolite levels. Instead, we examined putative metabolomic phenotypes specific to either SDHRCC or FHRCC as identified by prior in vitro studies. Consistent with prior reports suggesting that excess fumarate can disrupt normal flux in the urea cycle [28,29], we found that several metabolites in the urea cycle were specifically elevated in FHRCC but not SDHRCC (Fig. 2D) when compared to normal kidney tissue, including argininosuccinate (log_2_ fold-change 1.21, *p* = 0.06 in FHRCC; log_2_ fold-change −0.96, *p* = 0.34 in SDHRCC) and citrulline (log_2_ fold-change 1.94, *p* = 0.03 in FHRCC; log_2_ fold-change 0.80, *p* = 0.10 in SDHRCC). Other urea cycle metabolites did not show significant changes in abundance (Fig. 2E).

### 3.5. Differential metabolic signatures can identify TCA cycle deficiency in a patient with an inconclusive diagnosis

**Fig. 3 –:**
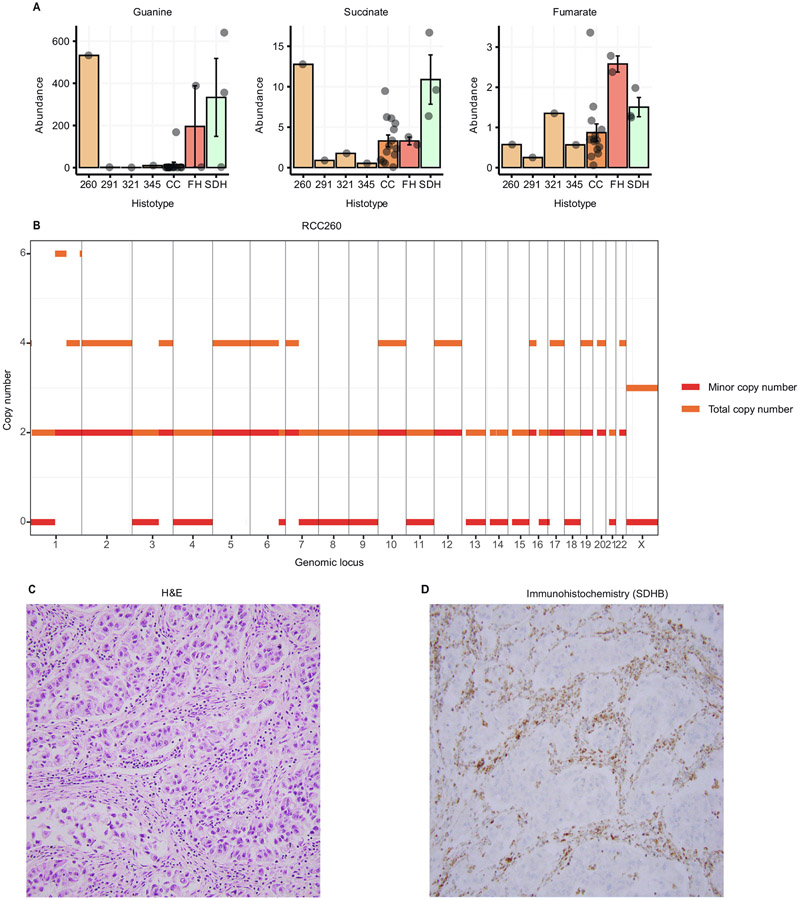
Metabolomic comparison of unknown sample with FHRCC and SDHRCC. Barplot of levels of guanine, succinate, and fumarate for four unclassified RCC samples (260, 291, 321, 345) and ccRCC, FHRCC, and SDHRCC tumors. Sample 260 demonstrated extreme elevation of guanine and succinate, without elevation of fumarate. This resembles the metabolic profile of other SDHRCC tumors. (B) Copy number profile of sample RCC260. Orange indicates total copy number and red indicates the minor copy number. Note the copy-neutral loss of heterozygosity on chromosome 1p, the locus of the *SDHB* gene. (C) Representative hematoxylin and eosin (H&E) image of tumor RCC260 showing infiltrating tubules and nests of neoplastic cells with high-grade nuclear features, eosinophilic cytoplasm, and scattered cytoplasmic vacuoles. (D) SDHB immunohistochemical stain of RCC260 shows a loss of SDHB protein expression in the neoplastic cells, whereas the stain is retained in the stromal, endothelial, and inflammatory cells.

As noted earlier, four uRCC tumors included in our metabolomics data clustered closely with the SDHRCC and FHRCC tumors, but separately from ccRCC on PCA (Fig. 2A). Although this suggests that some uRCCs may metabolically resemble non–clear-cell RCC histologies such as SDHRCC and FHRCC, we also considered the possibility that some of the uRCC tumors included in this small cohort may in fact harbor an overlooked loss of FH or SDH [42]. Reasoning that idiopathic accumulation of guanine, in combination with succinate and fumarate, strongly distinguished SDHRCC/FHRCC from ccRCC, we investigated the levels of these metabolites in the four uRCC samples. Only one tumor (RCC260) demonstrated elevation of guanine (log_2_ fold-change 5.30) and succinate (log_2_ fold-change 1.95), but not fumarate (log_2_ fold-change −0.60) when compared to ccRCC (Fig. 3A). This suggests that RCC260 may be SDHdeficient. For 3/4 uRCC tumors (excluding RCC345), we had matched MSK-IMPACT sequencing data. Of these, only RCC260 demonstrated arm-level loss of chromosome 1p (Fig. 3B), which was a universal event in SDHRCC.

Patient RCC260 was a 44-yr-old male who presented with a kidney mass measuring 14.6 cm on imaging along with multiple bone lesions. Genitourinary pathology review of a bone biopsy noted a high-grade uRCC subtype with suspicion of distal nephron differentiation. He was treated with first-line pazopanib and passed away 10 months later. Critically, this patient was among the first to consent to MSK-IMPACT sequencing, several years before SDHRCC and FHRCC were recognized as distinct entities. There was no evidence of somatic mutation of either *SDH* or *FH* via MSK-IMPACT testing. The germline status of FH and SDH subunits was unknown. Given the presence of 1p LOH and the characteristic metabolic features seen, we hypothesized that RCC260 may be a SDHRCC tumor. IHC analysis showed loss of SDHB protein expression in neoplastic cells, but retention in stromal, endothelial, and inflammatory cells (Fig. 3C, D). Genomic, metabolomic, and IHC evidence therefore suggests that RCC260 represents a SDHRCC case, probably with an unidentified germline alteration. Taken together, these data argue that metabolomic profiling and germline testing (especially in uRCC tumors) may provide valuable supportive evidence in the diagnosis of SDHRCC and FHRCC.

## 4. Discussion

Despite superficially similar pathophysiology, SDHRCC and FHRCC are genomically distinct entities. While SDHRCC appears to be mainly driven by biallelic loss of *SDHB* and CNAs, FHRCC demonstrates more genomic diversity. SDHRCC tumors had a lower TMB and lower FGA than FHRCC tumors. The comparative genomic stability of SDHRCC relative to FHRCC suggests that from an evolutionary perspective, SDHRCC tumors fail to genomically diversify and that biallelic loss of *SDHB* is sufficient to drive tumorigenesis.

Our data establish that SDHRCC and FHRCC arise through biallelic loss of *SDHB* or *FH* via one of two genetic mechanisms: either an initial germline mutation followed by a somatic second hit [40], or alternatively (and more rarely) purely somatic loss of both alleles. Prior work by our colleagues established that germline-associated FHRCC and purely somatic FHRCC were not distinguishable on the basis of morphology, IHC, patterns of disease spread, or response to systemic therapy (consistent with data for the overlapping samples presented here) [13]. In complement to this, we investigated the single case of purely somatic SDHRCC in our cohort, noting that the patient was among the oldest in our cohort (55 yr, consistent with the additional time required to accumulate two independent hits), but was otherwise molecularly indistinguishable. Interestingly, adjacent-normal kidney in both FHRCC and SDHRCC was metabolomically indistinguishable from adjacent-normal kidney in ccRCC, indicating that both *FH* and *SDHB* are haplosufficient and require two hits to produce metabolomic changes. Because of the *n*-of-1 nature of the purely somatic SDHRCC case, it is difficult to conclude if purely somatic SDHRCC is molecularly or clinically distinguishable from SDHRCC in patients with germline mutation, and these results should motivate future investigation into identifying phenotypic differences between these entities.

Because complete loss of SDH or FH disrupts the normally high metabolic flux through the TCA cycle, largescale changes to metabolism are expected [38]. There was a clear separation between SDHRCC/FHRCC tumors and ccRCC tumors. Although succinate was elevated in both SDHRCC and FHRCC compared to normal kidney tissue, fumarate was elevated exclusively in FHRCC. When SDHRCC/FHRCC tumors were compared to normal kidney, we observed extreme elevation of the purine derivative guanine. We suspect a mechanism unrelated to purine catabolism is involved, as there was no evident excess of xanthine, the catabolic breakdown product of guanine. This accumulation appeared to be pathognomonic and was evident in a suspected SDHRCC case found in our uRCC cohort.

As the field moves towards molecular stratification of RCCs [30], integration of metabolomic data may provide a new lens for distinguishing tumor subtypes and uncovering novel therapeutic targets. For example, it has been shown that accumulation of fumarate and succinate due to loss of enzyme function suppresses homologous recombination and renders tumor cells vulnerable to synthetic lethality with PARP inhibitors [31]. It has also been shown that FH and SDH regulate HIF-α prolyl hydroxylases, thereby leading to stabilization and activation of HIF-1α [32,33]. Similar to the development of and clinical activity seen with HIF2-α inhibitors [34], further study into the downstream effects of loss of enzyme activity may provide a mechanistic target for similar agents. Lastly, serum 2-hydroxyglutarate has been identified as a biomarker in peripheral blood [35,36] and via radiographic detection [37] for IDH-mutant tumors. Given the similar role of IDH in the TCA cycle, future studies that explore fumarate, succinate, or other TCA cycle metabolites may uncover novel circulating biomarkers in this patient population.

Our study has limitations inherent to studies on rare tumors. To increase our sample size, we included patients from an external multi-institutional cohort that had limited clinical information available. We acknowledge that there is heterogeneity in the type and depth of genetic sequencing and metabolomic analyses. Despite the statistical limitations and the heterogeneous data, our genomic and metabolomic analysis of FHRCC and SDHRCC provides insights into the defining molecular phenotypes of these entities and serves as a foundational resource for future investigation.

## 5. Conclusions

In conclusion, our sequencing and metabolomic analyses showed that despite sharing similar genetic etiology, SDHRCC and FHRCC represent distinct molecular entities with unique genetic and metabolic abnormalities.

## Supplementary Material

3

1

2

5

4

6

7

## Figures and Tables

**Table 1 –:** Demographic and clinical characteristics of the FHRCC and SDHRCC patients in our cohort

Parameter	Full cohort ^a^	
	FH (*n* = 25)	SDH (*n* = 17)
Median age at diagnosis, yr (range)	47 (20–74)	32 (14–68)
Sex, *n* (%)		
Female	9 (36)	5 (29)
Male	16 (64)	12 (71)
Race, *n* (%)		
Asian/Indian subcontinent	1 (4.0)	0 (0)
Black or African American	6 (24)	0 (0)
Unknown or refused to answer	3 (12)	9 (53)
White	15 (60)	8 (47)
Nephrectomy, *n* (%)	20 (80)	16 (94)
Kidney primary tumor size, *n* (%)		
≤4 cm	6 (24%)	2 (12%)
4–7 cm	4 (16%)	4 (24%)
>7 cm to ≤10 cm	5 (20%)	9 (53%)
>10 cm	10 (40%)	2 (12%)
Inclusion criteria, *n* (%)		
Genomic	22 (88%)	17 (100%)
Immunohistochemistry	3 (12%)	0 (0%)
Median time to metastasis, d (range)	421 (78–4134)	414 (414–414)
	**Memorial Sloan Kettering patients**	
	FH (*n* = 25)	SDH (*n* = 9)
Personal history of syndromic features, *n* (%)		
Cutaneous leiomyomas	1 (4.0)	0 (0)
None	18 (72)	6 (67)
Paraganglioma	0 (0)	1 (11)
Pheochromocytoma	0 (0)	2 (22)
Uterine fibroids	4 (16)	0 (0)
Uterine fibroids, cutaneous leiomyomas	2 (8.0)	0 (0)
Family history of syndromic features, *n* (%)		
Kidney cancer	2 (8.0)	3 (33)
Kidney cancer, uterine fibroids	1 (4.0)	0 (0)
None	14 (56)	6 (67)
Uterine fibroids	7 (28)	0 (0)
Uterine fibroids, cutaneous leiomyomas	1 (4.0)	0 (0)
Extent of disease at initial RCC diagnosis, *n* (%)		
Distant metastases	13 (52)	3 (33)
Localized	12 (48)	6 (67)

RCC = renal cell carcinoma; FHRCC = FH-deficient RCC; SDHRCC = SDF-deficient RCC.

a There were limited clinical data for patients from external institutions.
