# Supplementary material for: Genomic and Metabolic Hallmarks of SDH- and FH-deficient Renal Cell Carcinomas
Source: Eur Urol Focus. Author manuscript; Available in PMC 2022 Dec 6. (PMC9464266; doi:10.1016/j.euf.2021.12.002)
Supplement: 1 [file NIHMS1788509-supplement-1.docx]

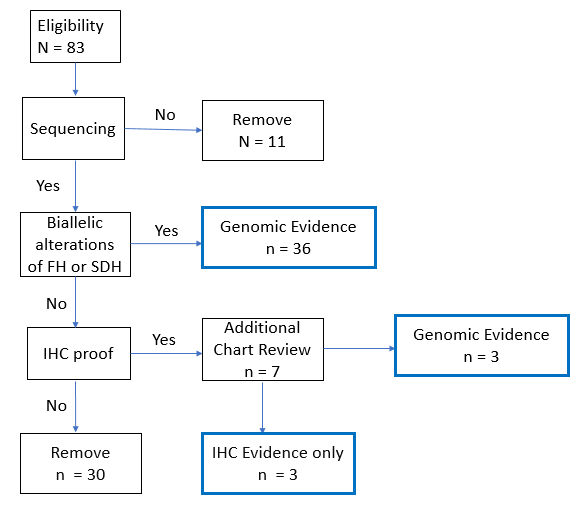


**Supplementary Figure 1:** 83 RCC patients with a presumed diagnosis of FHRCC or SDHRCC were identified using a combination of genomic, immunohistochemical (IHC), and expert genitourinary pathology review. Of these, 11 patients were excluded due to lack of sequencing data either through MSK-IMPACT, WES or WGS. Patients who demonstrated biallelic alterations of *SDHA/SDHB/SDHC/SDHD/SDHAF2* or *FH* were considered to have genomic evidence of disease. 36 patients demonstrated biallelic loss of the gene of interest. For the remaining patients, immunohistochemical staining was evaluated for loss of protein expression (FH or SDH). 6 patients demonstrated loss of FH or SDH on IHC, prompting additional chart review for genomic evidence of disease. Of the 6 that were evaluated, 3 patients were identified as having biallelic alterations due to genetic testing either at an outside institution or through a different clinical genetic service. 3 patients IHC evidence of disease only. The final cohort consisted of a total of 42 patients, 25 patients were FH-deficient and 17 patients were SDH-deficient.

**Supplementary table captions**

**Supplementary Table 1.** Clinical, Pathologic, and Molecular Features of TCA cycle -deficient Renal Cell Carcinomas.

**Supplementary Table 2.** Summary of systemic therapies received by SDHRCC patients.

**Supplementary Table 3.** Mutations (germline and somatic) occurring in the SDHRCC/FHRCC cohort.

**Supplementary Table 4.** Copy number information from FACETS output for samples in SDHRCC/FHRCC cohort.

**Supplementary Table 5:** Related to Figure 2. Results of differential abundance tests for metabolites in this study, comparing SDHRCC/FHRCC to normal kidney, ccRCC, and each other.

**Supplementary Table 6:** Results of differential abundance tests for metabolites in this study, comparing a multi-regional FHRCC tumor to its adjacent normal kidney tissue.
